# Revealing Temozolomide Resistance Mechanisms via Genome-Wide CRISPR Libraries

**DOI:** 10.3390/cells9122573

**Published:** 2020-12-01

**Authors:** Clarissa Ribeiro Reily Rocha, Alexandre Reily Rocha, Matheus Molina Silva, Luciana Rodrigues Gomes, Marcela Teatin Latancia, Marina Andrade Tomaz, Izadora de Souza, Linda Karolynne Seregni Monteiro, Carlos Frederico Martins Menck

**Affiliations:** 1 Department of Clinical and Experimental Oncology, Federal University of São Paulo (UNIFESP), São Paulo 04037-003, Brazil; clarissa.rocha@unifesp.br (C.R.R.R.); marina.tomaz@unifesp.br (M.A.T.); izadora.souza@unifesp.br (I.d.S.); linda.seregni@unifesp.br (L.K.S.M.); 2 Institute of Theoretical Physics, State University of São Paulo (UNESP), São Paulo 01140-070, Brazil; alexandre.reily@unesp.br; 3 Department of Microbiology, Institute of Biomedical Sciences, University of São Paulo (USP), São Paulo 05508-000, Brazil; matheusmolina95@gmail.com (M.M.S.); marcela.latancia@gmail.com (M.T.L.); 4 Laboratory of Cell Cycle, Center of Toxins, Immune Response and Cell Signaling (CeTICS), Butantan Institute, São Paulo 05503-001, Brazil; luciana.gomes@butantan.gov.br

**Keywords:** temozolomide, cancer resistance, glioblastoma, CRISPR library, NRF2

## Abstract

Glioblastoma is a severe type of brain tumor with a poor prognosis and few therapy options. Temozolomide (TMZ) is one of these options, however, with limited success, and failure is mainly due to tumor resistance. In this work, genome-wide CRISPR-Cas9 lentiviral screen libraries for gene knockout or activation were transduced in the human glioblastoma cell line, aiming to identify genes that modulate TMZ resistance. The sgRNAs enriched in both libraries in surviving cells after TMZ treatment were identified by next-generation sequencing (NGS). Pathway analyses of gene candidates on knockout screening revealed several enriched pathways, including the mismatch repair and the Sonic Hedgehog pathways. Silencing three genes ranked on the top 10 list (MSH2, PTCH2, and CLCA2) confirm cell protection from TMZ-induced death. In addition, a CRISPR activation library revealed that NRF2 and Wnt pathways are involved in TMZ resistance. Consistently, overexpression of FZD6, CTNNB1, or NRF2 genes significantly increased cell survival upon TMZ treatment. Moreover, NRF2 and related genes detected in this screen presented a robust negative correlation with glioblastoma patient survival rates. Finally, several gene candidates from knockout or activation screening are targetable by inhibitors or small molecules, and some of them have already been used in the clinic.

## 1. Introduction

Glioma is the most common primary cancer of the central nervous system, and approximately half of the patients present the most aggressive form of the disease, glioblastoma multiform [1,2]. Current standard-of-care includes surgery for tumor resection, radiotherapy, and concomitant adjuvant chemotherapy, but this has not resulted in significant improvements in survival outcomes, and patient prognosis remains dismal with a median survival rate of 15 months [3].

The alkylating agent temozolomide (TMZ) has received attention for being one of a few orally administered antitumor drugs and for its ability to easily cross the blood-brain barrier [4,5]. Thus, after FDA approval in 2005, TMZ became the first-line drug therapy to treat glioblastoma [6]. This drug acts by inducing several types of DNA lesions through methylation. One particular type of lesion known as O^6^-methylguanine (O^6^meG) is especially harmful to the cells. This lesion can be directly repaired by a suicide enzyme known as MGMT (O-6-methylguanine-DNA methyltransferase), which removes the methyl group of the DNA base and transfers it to its cysteine residue [1]. However, if such a lesion is not repaired, it can originate a DNA mismatch (O^6^meG:T) upon replication. Importantly, the DNA mismatch can be recognized by mismatch repair (MMR) pathway that removes a patch of the newly synthesized strand, leaving O^6^meG into the original strand, leading in turn to another O^6^meG:T mismatch. This process of recognition, removal, and misincorporation of thymine occurs on S phase of cell cycle and can lead to replication fork collapse, and, as a consequence, cell death induction.

However, glioblastoma resistance towards TMZ-induced cell death severely limits the drug’s long-term efficacy. Several TMZ resistance mechanisms were described, but many more remain to be uncovered [7,8]. Hence, to develop glioblastoma therapies of clinical relevance, it is imperative to understand better the mechanisms of TMZ resistance and design novel combination therapies to increase efficacy and prevent tumor recurrence.

Anticancer drug resistance is a multifactorial phenomenon that may include: decreased drug uptake, increased drug efflux, enhanced drug detoxification, enhanced DNA repair capacity, molecular alterations of drug targets, and epigenetic changes [9]. Thus, a full understanding of TMZ resistance, associated with approaches to overcome it, is urgent and necessary to improve the efficacy of therapy protocols.

Comprehensive approaches (such as genome-wide screening) are required to understand the elements that lead to drug resistance. The conventional way to identify and validate drug-resistant genes classifies them in two groups: loss-of-function (e.g., RNAi) and gain-of-function (e.g., cDNA-based over-expression). Although these approaches have played essential roles in many significant discoveries in cancer biology over the past decades, they have some crucial limitations. cDNA-based expression systems may bring to supraphysiological levels of gene expression, causing artifact effects on cell biological processes [10]. Moreover, knockdown of a gene of mRNA levels by RNAi is incomplete, and the remaining mRNA molecules may still play a functional role, preventing identification of targets that require mRNA complete inactivation [11].

Thus, a high-throughput approach, which pinpoints most of the mechanisms involved, requires a significantly different methodology. Recently, CRISPR-Cas9 knockout (CRISPRKO) and CRISPR activation (CRISPRa) sgRNA libraries have made the entire genome amenable for loss-of-function and gain-of-function screens [12]. Due to its simplicity and efficiency, CRISPR functional screens have rapidly been adopted in various contexts and can revolutionize drug discovery and therapy [13]. This novel type of genomic screening has been applied in several tumor cell lines to identify essential genes or genes involved in drug response [14,15,16].

Using CRISPRKO and CRISPRa screens, several novel gene candidates and cellular pathways were identified and validated as related to drug resistance in human glioblastoma cells. U138MG glioma cells were transduced with CRISPR libraries and treated with sub-lethal doses of TMZ for two weeks. Next-generation sequencing (NGS) uncovered genes enriched after depletion or activation upon selection of resistant cells to TMZ treatment. Data analyses revealed two lists of genes whose absence or activation contributed to TMZ resistance. Notably, besides detecting expected hits, several novel genes and cellular pathways—such as circadian rhythm and Wnt pathways—were identified in the CRISPRKO and CRISPRa libraries. Some of these genes were tested and confirmed their protective action to TMZ, thus revealing potential novel mechanisms of glioma cell resistance to this chemotherapeutic agent.

## 2. Materials and Methods

### 2.1. Cell Lines and Culture Conditions

Human glioblastoma cell line U138MG was kindly provided by Prof. Bernd Kaina, Mainz University, Germany. U138MG was routinely grown in DMEM (Invitrogen, Life Technologies, Carlsbad, CA, USA), supplemented with 10% fetal calf serum (FCS; Cultilab, Campinas, SP, Brazil) and 1% antibiotic at 37 °C in a humidified, 5% CO_2_ atmosphere.

### 2.2. Pooled Genome-Wide CRISPR Screen

Genome-scale CRISPR knockout library preparation and lentivirus production Genome-scale Human CRISPR Knock-Out (GeCKO) (1000000048; Addgene, Watertown, MA, USA) library and Human CRISPR activation library (SAM) (1000000057 Addgene) were amplified using Endura electrocompetent Escherichia coli cells (60242; Lucigen, Middleton, WI, USA). To prepare lentiviral particles, the plasmids of the CRISPR libraries were co-transfected with plasmids pMD2.G (Addgene, cat. no. 12259) and psPAX2 (Addgene, cat. no. 12260) into HEK 293T cells. Lentiviral were harvested 72 h later, concentrated by ultracentrifugation at 19,600 rpm for 2 h at 4 °C, resuspended in DMEM, and stored at −80 °C. U138MG-Cas9, and U138MG-VP64/P65 cells were infected with lentiviral libraries pooled GeCKO v2 and SAM libraries, respectively, at a multiplicity of infection of 0.3. After 5 days of puromycin (GeCKO v2) or zeocin (SAM) selection, 5 × 10^7^ cells were treated with 0.2% DMSO (vehicle) or 100 μM TMZ for an additional 2 weeks. PCR was performed on genomic DNA to construct sequencing libraries with each containing around 300 μg DNA each. High Fidelity Q5 polymerase (New England Biolabs, Ipswich, MA, USA) was used to amplify the sgRNA cassette from the isolated genomic DNA templates. PCR amplicons were gel-purified and subjected to next-generation sequencing using NextSeq 500 (Illumina, San Diego, CA, USA). The experiments were independently performed twice. Each library was sequenced at around 10–20 million reads to achieve 100× average coverage over the CRISPR library.

### 2.3. Computational Analysis of the Screens

The CRISPR/Cas9 screening data were processed and analyzed using the MAGeCK-VISPR algorithm. The MAGeCK-VISPR collected read counts of all sgRNAs in all conditions from fastq files, and then normalized the read counts of control and treatment conditions using median normalization. MAGeCK-VISPR built a linear model to estimate the variance of sgRNA read counts, evaluated the sgRNA abundance changes between control and treatment conditions, and assigned a *p* value using the negative binomial. Finally, the selection of genes is evaluated from the rankings of sgRNAs (by their *p* values). A detailed description of the MAGeCK algorithm can be found in the original study [17].

### 2.4. Cell Survival Measurement

In a 12 multi-well plate, 2 × 10^4^ cells were plated and the next day treated with increasing doses of TMZ for 72 h. After that, the cells were washed with phosphate-buffered saline (PBS) followed by incubation with the XTT reagent kit as recommended by the manufacturer’s instructions (Roche, Basel, Switzerland).

### 2.5. Gene Silencing

The esiRNA (40 nM final concentrations) targeting human MHS2, CLCA2, PTCH2, and EGFP—as control—were purchased from Sigma Aldrich (Saint Louis, MO, USA). Transfections were performed using Lipofectamine RNAiMAX Reagent (Invitrogen) following the manufacturer’s instructions. siRNAs were incubated for 24 h prior to TMZ treatment.

### 2.6. Establishing Cell Line Overexpression Target Genes

Lentivirus containing cDNA NFR2, FZD6, and CTNNβ1 were purchased from Vector Builder. The plasmids of interest were co-transfected with three auxiliary plasmids into HEK 293FT cells using the polyethyleneimine (PEI) transfection method. The recombinant lentivirus was individually used to transduce U138MG, followed by puromycin selection, resulting in cells stable-expressing NRF2, FZD6, CTNNβ1, or with an empty vector (as control).

### 2.7. Real-Time PCR

Total RNA was extracted using PureLink RNA Mini kit (Invitrogen), following the manufacturer’s protocol. After DNase (Promega, Madison, WI, USA) treatment, cDNA was prepared using a High Capacity cDNA Reverse Transcription kit (Applied Biosystems, Life Technologies, California, CA, USA). Real-time quantitative PCR (RT-PCR) determined gene expression. Briefly, 3 μL of diluted cDNA, 6 μL of SYBR green master mix, 0.5 μL of 10 mM forward and reverse primers, and nuclease-free water were used in a combined total volume of 12 mL for each reaction. RT-PCR was carried out using the 7500 Real-Time PCR System (Applied Biosystems). The relative expression levels of the genes of interest were calculated using the relative standard curve method, based on the individual RT-PCR primer efficiencies, and the quantified values were normalized against the housekeeping gene encoding GAPDH.

### 2.8. Cell Migration

The cell migration assay was performed as previously described [18]. Briefly, migratory ability of U138MG cells was assessed using a transwell chamber (6.5 mm insert diameter, 8 μm pore size) placed in 24-well culture plates. U138MG overexpressing either FZD6, CTNNβ1, or transduced with control vector (5 × 10^4^ per well) were suspended in 10% FCS DMEM and seeded to the upper chambers of the transwell plates, and the lower chambers were filled with 1 mL of DMEM containing 20% FCS. Following 36 h of incubation at 37 °C, cells located on the upper membranes were removed with Q-tips, and the cells that migrated through membranes were fixed with 95% methanol and stained with 0.1% crystal violet solution for 15 min. Images of the stained cells that migrated to the lower sides of the filter were captured with an inverted microscope.

### 2.9. Bioinformatics

To assess the relationship between NRF2 target genes expression and patients’ risk/survival in different human glioblastoma samples, the SurvExpress tool (28) was used. The SurvExpress database comprises microarray gene expression and matched clinical data of several tumors. The TGCA glioblastoma multiforme (538 patients) sample was used. For the input gene list of NRF2 targets (GSK3B, UBE2E3, UBE2E1, GSTP1, NFE2L2, SQSTM1, MAFB, HMOX1, SIAH2, GSTM4, SRXN1, CASP3, GNAI1, COL3A1, ABCC3, PLEKHH2, GCLM, SLC7A2), the software calculates the prognostic index (PI), also known as the risk score, for each sample. PI values were used to split samples and generate 2 risk groups (low and high risk); Overall Survival was used as a clinical endpoint. Quartile normalized values of probe expression were used, and, for multiple probe genes, the maximum variance probe was selected. Log-rank test of differences between risk groups, hazard-ratio (HR) estimate, and gene expression fold-changes in high and low-risk groups were calculated in order to identify the NRF2 targets with differential expression. These fold-change values were used to estimate an NRF2 Pathway Signature Expression in high-risk versus low-risk patients in the TCGA glioblastoma cancer dataset.

### 2.10. Statistical Analysis

Excepting from CRISPR libraries genomic screenings—which were performed twice—all the results represent the mean of three independent experiments, each performed in triplicate, with error bars showing the standard error of the mean (SEM). Statistical significance among datasets was accessed by applying one-way ANOVA followed by Bonferroni post-testing (Prism 6—GraphPad Software Inc., CA, USA) (* *p* < 0.05, ** *p* < 0.01, *** *p* < 0.001).

## 3. Results

### 3.1. Genome-Wide CRISPR/Cas9 Knockout Screen in Glioblastoma Cancer Cell

The main goal of this work is to identify genes involved in TMZ resistance promotion on glioblastoma cells. As a first approach, a pooled genome-wide CRISPR/Cas9 knockout screen, in Cas9 constitutively expressing U138MG cells, was performed with the GeCKO v2 lentiviral single guide RNA (sgRNA) library [15]. This loss-of-function library contains 123,411 unique sgRNAs targeting 19,050 genes, with six sgRNAs per gene. The sgRNAs integrated into the cellular genome with the lentivirus vector for their expression and editing of target genes. After selection with puromycin, half of the infected cells were continuously cultured for an additional two weeks with 100 μM TMZ or 0.2% DMSO (control sample). Surviving cells had their integrated sgRNAs amplified from genomic DNA and subjected to NGS sequencing for quantification, and data analyzed with the MAGeCK-VISPR tool [17].

The results revealed 1071 significant differentially enriched sgRNAs predicted to target 251 endogenous genes (*p* < 0.01) in the TMZ sample when compared to the control sample (the CRISPRko list in Supplementary Table S1). As it can be seen in Table 1, sgRNA read counts in the control and treatment biological replicate samples are quite similar. Furthermore, among the top 25 most enriched sgRNA, there are several targeting the same individual gene. In summary, this consistency indicates that our results are reliable.

**Figure 1:**
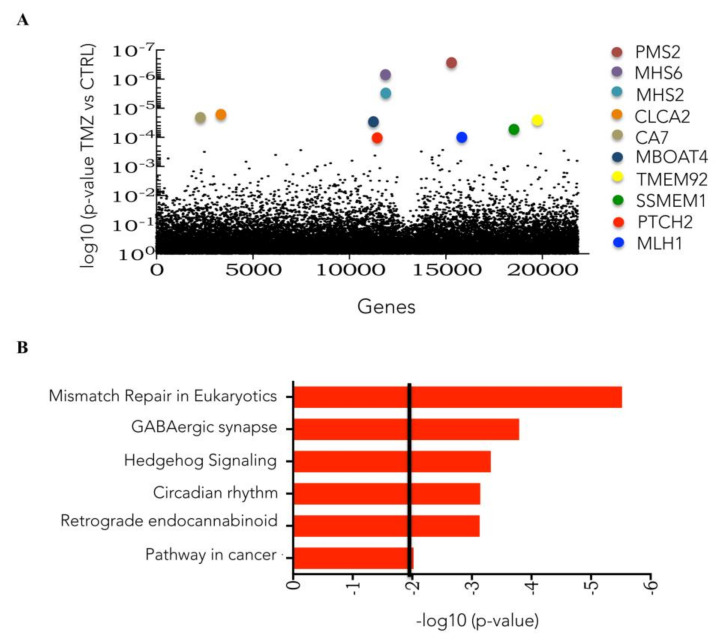
Identification of TMZ-resistant gene candidates and pathways with the knockout library. (**A**) Plot of enriched CRISPR Knock-Out (GeCKO) library individual sgRNA in the TMZ-treated samples compared to vehicle (DMSO) samples determined by MAGeCK-VISPR tool analysis. (**B**) Cellular pathway enrichment of TMZ treated samples compared to DMSO treated samples as determined by DAVID tool analysis.

Figure 1A shows the top 10 genes in this list. Using the DAVID tool, pathways enriched in cells surviving TMZ were detected. Among these, previously known TMZ resistance-related pathways, such as MMR, and novel pathways, such as those related to the Sonic Hedgehog signaling and the circadian rhythm, were also identified (Figure 1B).

### 3.2. CRISPRKO Screen Gene Candidates Validation

**Figure 2:**
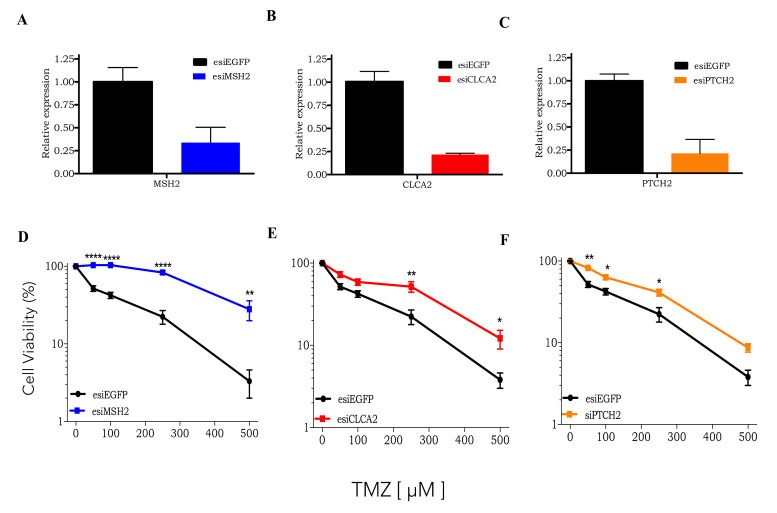
Validation of knockout library gene candidates**.** (**A**–**C**) RT-PCR of MSH2, CLCA2, and PTCH2 genes in U138MG cells transfected with esiEGFP or esi of gene of interested. (**D**–**F**) Dose-response curves of U138MG cells submitted to gene silencing (MSH2, CLCA2, PTCH2, or EGFP), treated with increasing concentrations of TMZ (50 to 500 µM) and analyzed after 120 h treatment using the XTT assay. * *p* < 0.05, ** *p* < 0.01, **** *p* < 0.0001.

Next, three of the top gene candidates identified by CRISPRKO screening were further tested for validation: MSH2 (DNA repair protein involved in MMR); CLCA2 (type 1 transmembrane protein that inhibits the Wnt pathway); PTCH2 (transmembrane protein that inhibits Sonic Hedgehog pathway). For this purpose, these genes were silenced using a siRNA approach (Figure 2A–C). In Figure 2D–F, all individually silenced genes upon TMZ treatment resulted in a significant increase in cell viability compared to control cells (esiEGFP). These results strongly confirm that, first, CRISPR library genomic screening is a powerful tool to identify genes related to TMZ resistance. Secondly, the activation of either Wnt or Sonic Hedgehog pathways may enhance tumor cells’ resistance to TMZ treatment.

### 3.3. Genome-Wide CRISPR/Cas9 Activation Screen in Glioblastoma Cancer Cell

CRISPRa screens enable rapid gain-of-function analysis at the genome-scale. For this screen, U138MG cells expressing transcriptional activation genes (VP64 and P65) were obtained with lentivirus dCAS-VP64_Blast and lenti-MS2-P65-HSF1_Hygro [16]. These cells were transduced with CRISPRa library, which targets the 200 bp region upstream of the transcriptional start site of 23,430 human coding isoforms with three sgRNAs per gene—a total of 70,290 unique sgRNAs—prioritizing sgRNAs with minimal off-target activity [16].

The cells were treated with TMZ (100 μM) or 0.2% DMSO (control) for two weeks, and surviving cells had their integrated sgRNA sequences amplified and sequenced by NGS. The results revealed 801 significant differentially enriched sgRNAs predicted to target 308 endogenous genes (*p* < 0.01) in the TMZ sample, compared to control cells (the list in Supplementary Table S2). As it can be observed in Table S2, for the CRISPRa library, most of the targeted genes were detected with more than two, and most with more than three sgRNA (in the table: last column—pos/goodsgrna). As displayed in Table 2, most of the top 25 most enriched sgRNA targeting a specific gene appeared more than once, and the sample read counts between biological replicates are quite consistent. This certainly reinforces the confidence of the data screened.

**Figure 3:**
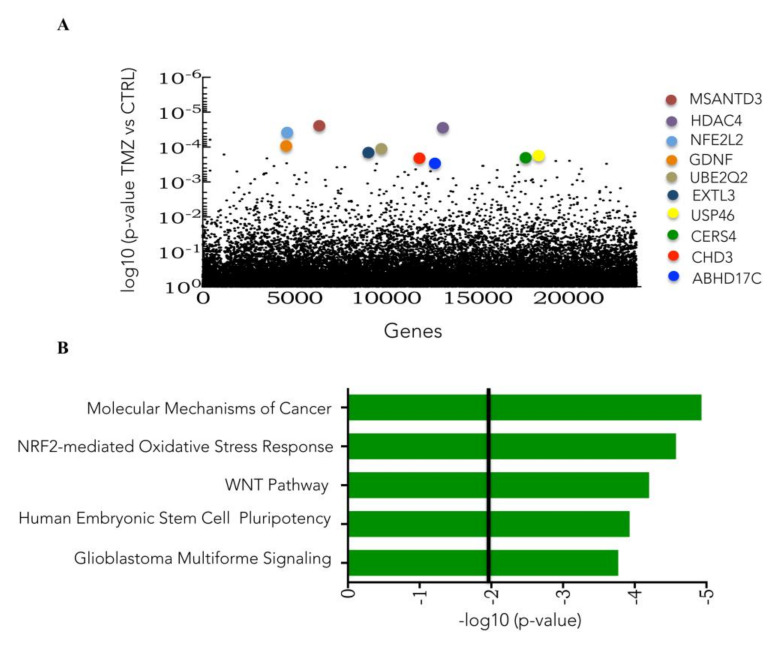
Identification of TMZ resistance gene candidates and pathways as determined by CRISPR activation library screening. (**A**) Plot of enriched Human CRISPR activation library of individual sgRNA in the TMZ treated samples compared to vehicle (DMSO) samples determined by MAGeCK-VISPR tool analysis. (**B**) Pathway enrichment analysis, performed by Ingenuity software, of TMZ-enriched genes given by CRISPR activation library screening analysis.

Figure 3A highlights the top 10 genes identified in the CRISPRa screen. The Ingenuity software detected the pathways enriched in TMZ surviving cells, revealing that NRF2, Wnt, and pluripotency were among them (Figure 3B).

### 3.4. CRISPR Activation Screen Gene Candidates Validation

The Wnt signaling pathway regulates a group of evolutionarily conserved genes that participate in a diverse set of cellular activities including cell proliferation, calcium homeostasis, and cell polarity. This pathway is subdivided into the canonical Wnt (or β-catenin-dependent) and the non-canonical Wnt (or β-catenin-independent) routes, and both have genes in the CRISPRa list (Supplementary Table S2). As one can easily see, many genes that play central roles in the Wnt signaling pathway are present in the CRISPRa gene list (such as, FZD6, CNNTβ1, and Wnt3a).

**Figure 4:**
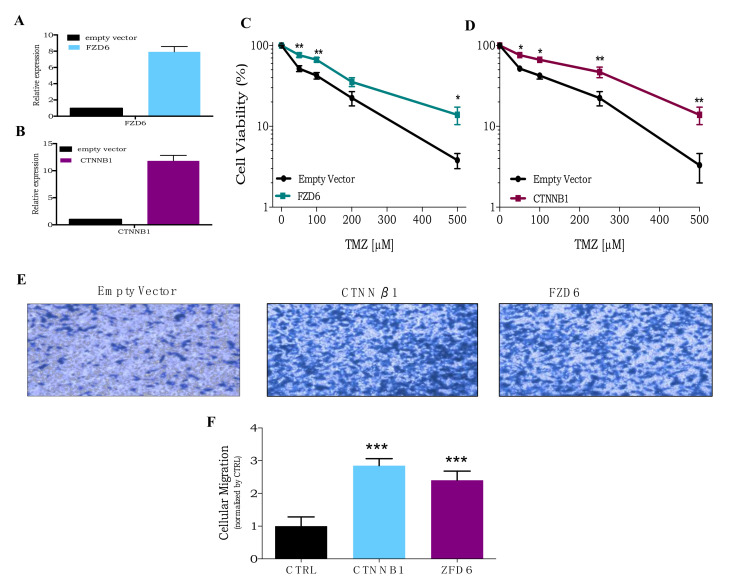
Overexpression of Wnt pathway genes increases TMZ resistance. (**A**,**B**) RT-PCR of FZD6 or CTNNB1 genes in U138MG transduced with empty vector, FZD6, and CTNNB1 lentivirus, respectively. (**C**,**D**) A dose-response curve of U138MG cells transduced with empty vector, CTNNB1, or FZD6 lentivirus (respectively), treated with increasing concentrations of TMZ (50 to 500 µM) and analyzed after 120 h treatment using the XTT assay. (**E**,**F**) Representative and quantification image, respectively, of cellular migration assay of U138MG overexpressing CTNNB1 or FZD6 and compared to empty vector as control. * *p* < 0.05, ** *p* < 0.01, *** *p* < 0.001.

Two key genes for Wnt signaling activation were chosen for validation: membrane receptor Frizzled (FZD6) and β-catenin (CNNTβ1). For this purpose, U138MG cell lines overexpressing either FZD6 or CNNTβ1 were established using lentivirus vectors (Figure 4A,B). As shown in Figure 4C,D, the cell lines overexpressing these genes (U138MG-FZD6 and U138MG-CNNTβ1) were less sensitive to TMZ cytotoxicity, when compared to control cells (carrying the empty vector). Moreover, both also show a significantly higher cellular migration capability than the control cell line (Figure 4E,F). These data indicate that activation of the Wnt pathway in glioblastoma cells promotes tumor aggressiveness and TMZ resistance.

### 3.5. Roles of NRF2 on TMZ Resistance

The transcriptional factor NRF2 or NFE2L2 (Nuclear factor erythroid-derived factor 2-related factor 2) is a well-known master regulator of antioxidant responses [19]. Under physiological conditions, NRF2 availability and function are tightly regulated by KEAP1 (Kelch-like ECH associated protein 1). KEAP1 binds to NRF2, leading to proteasome degradation. However, in an oxidative stress situation, KEAP1 is oxidized, and NRF2 is phosphorylated by PKC, inducing nuclear NRF2 translocation and the activation of antioxidant responsive elements (ARE). More than 100 genes contain ARE sequences within their promoters and are therefore under the control of the NRF2 protein [20]. Among these genes, NRF2 controls the expression of enzymes GCL and GCLC responsible for GSH synthesis and also enzymes related to GSH utilization such as glutathione reductase, glutathione peroxidase, and glutathione transferases (GST) [21].

**Figure 5:**
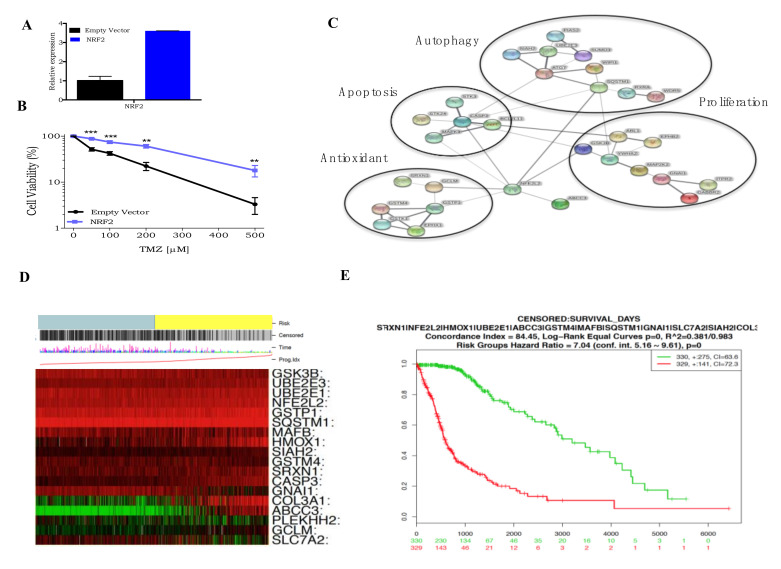
NRF2 mediates TMZ resistance and predicts glioblastoma patients’ survival. (**A**) RT-PCR of NRF2 gene in U138MG transduced with empty vector or NRF2 lentivirus. (**B**) A dose-response curve of U138MG cells transduced with empty vector and NRF2 lentivirus treated with increasing concentrations of TMZ and cell viability analyzed after 120 h treatment using the XTT assay. (**C**) NRF2 interaction network (as determined by STRING) displaying NRF2 interaction with target proteins identified on activation gene candidates list. (**D**) Heatmap representation of NRF2 target genes expression (green indicates low expression, while red indicates low expression), and its correlation with low risk and high risk—grey or yellow bar in the figure top—in the TCGA glioblastoma tumors as determined by the SurvExpress tool. (**E**) Kaplan-Meier curve showing the impact of NRF2 pathway expression signature upregulation (red) or downregulation (green) in glioblastoma cancer patients’ survival as determined by the SurvExpress tool. ** *p* < 0.01, *** *p* < 0.001.

Confirming NRF2 confers resistance to TMZ, a U138MG cell line overexpressing NRF2 (U138MG-NRF2), constructed with a lentivirus vector (Figure 5A), displayed significantly higher cellular survival upon TMZ treatment than control cells (U138MG carrying the empty vector) (Figure 5B). As mentioned above, NRF2 modulates the expression of dozens of genes, so all the known NRF2 regulated genes present in the CRISPR activation screen list were selected and used to build an NRF2 interaction network map given by the STRING software (Figure 5C). The result shows that several of the NRF2 related genes in this list are implicated in different cellular pathways highly related to carcinogenesis, such as cell proliferation, apoptosis, autophagy, and antioxidant responses.

The relevance of the NRF2 pathway activity directly on patients was examined using the data of TGCA glioblastoma tumor samples. Gene expression in tumor samples was classified by patient risk, in which green represents a low risk, and red represents a high risk. As shown in Figure 5D, the higher expression of the NRF2 gene and related genes in glioblastoma tumors, the higher the risk for the patients. These results can be visualized more clearly in a Kaplan-Meier plot of overall survival rate of glioblastoma patients related to NRF2-related gene expression (Figure 5E). These results strongly indicate that NRF2-related gene expression can predict glioblastoma patient prognosis.

**Figure 6:**
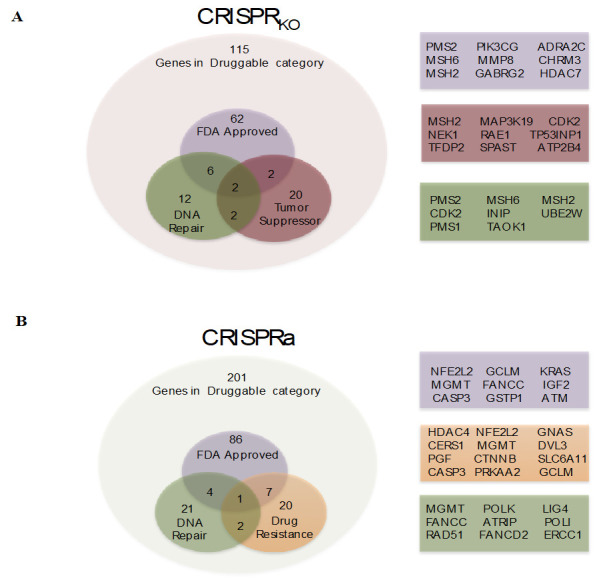
Identification of druggable genes. (**A**) Identification of activation CRISPR library screen gene candidates that fit in druggable category and sub-category of FDA-approved; DNA repair; drug resistance given by DGIdb tool (http://www.dgidb.org/). (**B**) Identification of knockout CRISPR library screen gene candidates that fitted in druggable category and sub-category of FDA-approved; DNA repair; tumor suppressor given by DGIdb tool.

Finally, to further identify genes that are potentially targeted for therapy, the CRISPRko and CRISPRa gene lists were submitted to the drug-gene interaction database (DGIdb) software analysis. This software is a powerful tool that can identify the so-called “druggable genes”. This category of genes has pharmacological modulators (inhibitor or activation) available, and more importantly, several of them have already been approved by the Food and Drug Administration (FDA). The results shown in Figure 6 indicate that out of 215 genes from the KO and 301 genes from the activation lists, 96 and 129 genes, respectively, were identified as druggable. For the KO library, 22 of these druggable genes have DNA repair functions, and 26 are tumor suppressors (four in common with DNA repair). For the activation library screen, 21 druggable genes have DNA repair functions, and 20 are classified as drug resistance genes (three in common with DNA repair). Therefore, among the genes identified as potentially linked to TMZ resistance, a considerable number of them can be targeted by available drugs. They may be used in combination with TMZ to potentiate tumor cytotoxicity. Importantly, the results also show that FDA approved drugs can modulate 86 and 62 genes identified in the activation or knockout gene lists, respectively.

## 4. Discussion

As with many cancers, anti-cancer therapeutic agents have not significantly increased the median survival of glioblastoma patients over the past ten years [22,23]. The 5 year survival rate of glioblastoma patients is low as 6.8%, even after treatment that includes surgical resection, radiation, and chemotherapy [23]. This low survival rate is a colossal failure, partially attributed to drug resistance [2]. Thus, identifying the cellular and molecular mechanisms that confer drug resistance is an essential goal for the treatment of glioblastomas.

New approaches are needed to systematically explore TMZ resistance in glioblastoma and develop clinically translatable and effective strategies. Here, CRISPR-Cas9 knockout and activation screen libraries led to the identification of novel genes and pathways, candidates for resistance mechanisms. With the CRISPR knockout screen library, the MMR pathway was confirmed as the most enriched pathway in TMZ surviving cells, with four MMR genes appearing as the top ten candidates detected (PMS2, MSH6, MSH2, and MLH1). As TMZ is a methylating agent on DNA, this repair pathway is known to participate directly on the cell death mechanism, by generating double-strand breaks on the DNA molecule [24]. Mismatch repair defective cells are more resistant to methylating agents [25]. Thus, the finding of MMR mutated cells resistant to TMZ in the CRISPR genome-wide screen was expected [26].

To this date, only two previous works used a CRISPR screening library approach to search for a TMZ resistance gene candidate. First, a recent work using a different CRISPRko library in glioblastoma stem cells treated with TMZ also revealed MMR genes as top gene candidates [27], and more recently, using U87MG and U87MG, EGFRvIII cells revealed that E2F6 controls TMZ resistance in EGFR mutates cells [28]. Notably, in the experiments reported here, a more extensive CRISPRko library was used, and, importantly, the TMZ dose is more similar to those employed in clinical procedures [29,30]. As a result, novel or poorly explored pathways were also identified as responsible for TMZ resistance, such as Sonic Hedgehog and circadian rhythm.

During organism development, the Sonic Hedgehog (Shh) signaling pathway functions in cell differentiation, growth, and tissue polarity. In the context of cancer, this pathway is involved in tumor progression and invasion [31]. At the molecular level, the transcriptional factor GLI induces several genes involved in tumor progression, and the transmembrane proteins PTCH1 and PTCH2 act as pathway inhibitors [32]. It was recently shown that Shh impairment by inhibition of GLI significantly enhances TMZ cytotoxicity in glioblastoma cell lines [33,34]. In addition, in myeloproliferative neoplasms, the loss of PTCH2, and, consequently, Shh pathway activation, it was shown to improve tumor resistance to Fluorouracil (5-FU) treatment [35]. Here, PTCH2 was identified as one of the most enriched genes in the knockout gene list and, more importantly, confirmed by PTCH2 silencing, which protected cells from TMZ cytotoxicity induction.

Another novel pathway involved in TMZ resistance revealed by the CRISPRko library was the circadian rhythm. This pathway is an intrinsic, time-tracking system that enables organisms to anticipate environmental changes, allowing them to adapt their behavior, physiology, and metabolism to the appropriate daytime. A set of clock genes, switching on and off each other, defines the circadian rhythm of near 24 h. At the cellular level, the circadian clock prominently controls many fundamental cellular processes, such as xenobiotic metabolism [36], DNA repair mechanisms [37], and cell cycle progression [38]. Strikingly, clock genes also regulate NRF2, and more importantly, GSH expression significantly varies throughout the day [36]. In the CRISPRko list, clock genes such as PER1, PER2, and RORB were detected, indicating that blockage of circadian rhythm may lead to TMZ resistance. Thus, the role of the circadian cycle on TMZ resistance needs further investigation, with the potential to support the implementation of TMZ chronochemotherapy regimen for glioblastoma patients.

One of the genes ranked at the top 10 list was CLCA2, a type I integral transmembrane protein that modulates cells proliferation and differentiation through inhibition of β-catenin. CLCA2 can be induced by DNA damage [39]. It can also suppress migration and invasion in breast and colorectal cancer cell lines [40], acting, thus, as a tumor suppressor. Moreover, CLCA2 expression is usually downregulated in cancer cells [41]. In this work, CLCA2 silencing also resulted in cells’ increased viability after TMZ treatment (Figure 2B), indicating this is also an exciting target for clinical therapy for glioblastoma patients, when used in combination with TMZ.

In addition, the CRISPRa screen library led to the identification of activated genes that improve glioma cell resistance to TMZ treatment. To the best of our knowledge, this is the first time that the CRISPRa library was used to search for TMZ resistance mechanisms in glioblastoma cells. Ingenuity analysis of the CRISPRa gene list revealed several cellular and molecular pathways enriched upon TMZ treatment, among them: the Wnt and NRF2-mediated oxidative stress response pathways.

The Wnt signaling is involved in complex processes, such as embryonic development, stem cell maintenance, and tissue homeostasis [42]. The canonical Wnt/β-catenin pathway regulates cell fate, proliferation, and survival, while non-canonical Wnt pathway is more often associated with differentiation, cell polarity, and migration [43]. Interestingly, several of the genes that participate in Wnt-related pathways were detected as enriched in the activation screen. Consistent with these findings, activation of the canonical Wnt/β-catenin signaling cascade induces MGMT expression, and its inhibition augments the cytotoxic effects of TMZ [44]. Recently, it was shown that Wnt pathway may contribute to TMZ resistance through induction of autophagy [45] and that Wnt activation upon TMZ treatment is mediated by PI3K/Akt pathway [46].

Confirming the findings of the activation screen, overexpression of either canonical (CTNNβ1) or non-canonical (FZD6) genes of the Wnt pathway confers resistance to TMZ treatment in glioblastoma cells (Figure 4A,B). In addition, glioblastoma cells overexpressing these genes also show a greater migration capability, indicating higher aggressiveness (Figure 4C,D). Interestingly, CLCA2, an inhibitor of the Wnt signaling pathway, was also detected in the knockout library screen, further disclosing its relevance as a TMZ resistance mechanism. These results reveal that the Wnt pathway is a potential target for TMZ combinatorial therapy in glioblastoma patients.

The transcriptional factor NRF2 is known as a master regulator of antioxidant response in the cells. NRF2 regulates the expression of a large number of genes involved in the maintenance of intracellular redox homeostasis and xenobiotic detoxification [21,47]. Recently, NRF2 activity was shown to be involved in the autophagy process [48,49] as well as mesenchymal transition and invasion in several tumors including glioblastoma [50,51]. Emerging research has demonstrated that hyperactivation of the NRF2 pathway enables protection against chemotherapeutic agents, creating an environment that favors the survival of malignant cells [19,52,53].

Previous work indicated that a glioma TMZ-resistant cell line (U138MG) has a higher NRF2 expression than a TMZ-sensitive cell line (U87MG). Furthermore, NRF2 is induced upon TMZ treatment, helping the glioma tumor cells to resist this treatment [54]. Consistent with the in vitro results, where TMZ resistance correlated with GSH levels, GSH depletion by injection of BSO (GSH inhibitor) combined with TMZ significantly inhibited tumor progression of glioma and melanoma in vivo. These results indicate that NFR2 activity in regulating GSH availability with a decisive role in determining cell resistance to TMZ in glioblastoma cells [54].

Besides NRF2, among the gene candidates detected in the CRISPR activation screen library, several NRF2 targets were also associated with the promotion of TMZ resistance in glioblastoma cells, including genes involved in cellular proliferation, autophagy, and antioxidant defense. Interestingly, among antioxidant defense genes detected, GCML and glutathione-S-transferases (GSTP1, GSTM4), support previous data on the importance of GSH on TMZ resistance [54,55,56,57]. Moreover, several other genes detected in the activation screen were found to interact with NRF2, even in pathways other than antioxidation responses, confirming their relevance to TMZ resistance. Interestingly, the TCGA RNA expression dataset in glioblastoma tumors for NRF2 and its targets could predict the patients’ overall survival. These results indicate the importance of activation of the NRF2 pathway for drug resistance processes in glioblastoma.

The present work revealed a great number of genes and pathways involved in TMZ resistance in glioblastoma. In addition, even though our findings were based on a single cell line, it is important to highlight that several works have identified some of the pathways shown here to be involved in TMZ resistance in different glioblastoma cell lines [58,59,60,61]. Thus, we speculate that most of those novel genes/pathways that we have identified might also be important. Therefore, further studies using additional cell lines and in vivo models are necessary in order to validate these findings.

In addition, the results of the druggable gene list given by DGIdb greatly expand the combinatorial therapy for TMZ that could be tested initially in vitro and in pre-clinical studies using mice. Indeed, some of the pathways displayed in the present work as being related to TMZ resistance are being explored. In this sense, there have been recent works using bortezomib [62] (a proteasome inhibitor) or olaparib [63] (PAPR inhibitor) in combination with TMZ showing an increase in cytotoxicity. This is a strong indication that the ones we have identified are indeed suitable targets. On the other hand, combinatorial therapy of TMZ with oxaliplatin, venetoclax, or imatinib was poorly or not at all explored. This is obviously an extremely exciting route. Since the successful drug combinations could be offered to glioblastoma patients as an improved form of therapy, we plan to explore some of these drugs in a therapeutic regimen in future works.

Finally, due to drug resistance complexity, intrinsic and extrinsic resistance to TMZ comes not from a single primary factor, but also multiple cellular pathways. Since the efficacy of TMZ treatment is affected by resistance mechanisms, future therapies must consider alternative and combination therapy, attacking several survival mechanisms that protect the cancer cells from apoptosis and promote tumor growth. In that sense, the results shown here revealed novel druggable targets that potentially confer resistance to TMZ, and, thus, this knowledge may be constructive to design more efficient chemotherapy strategies to treat glioblastoma patients.

## Figures and Tables

**Table 1:** Top 25 CRISPRko library enriched sgRNA upon temozolomide (TMZ) treatment.

sgRNA	Gene	Control_count	Treatment_count	FDR	Rank
HGLibA_56959	PMS2	10.1/8.3	1644.2/1039.1	0	1
HGLibA_29380	MLH1	71.8/75.3	10,516.1/8493.7	0	2
HGLibB_45493	SLPI	62.3/60.9	1612.5/22,063.1	3.3 × 10^−283^	3
HGLibA_30203	MSH6	145.4/314.7	21,025.1/17,627.1	1.5 × 10^−135^	4
HGLibB_30160	MSH6	56.9/51.6	4275.1/3345.3	1.8 × 10^−154^	5
HGLibB_30150	MSH2	109.2/147.5	8836.2/8593.6	4.6 × 10^−100^	6
HGLibB_30148	MSH2	40.9/5.2	963.5/914.5	5.1 × 10^−240^	7
HGLibA_53112	UQCRC1	22.5/14.4	1063.1/995.6	2.2 × 10^−170^	8
HGLibB_53357	USPL1	21.4/16.5	1051.1/997.9	6.3 × 10^−153^	9
HGLibB_40798	REPS1	31.5/3.1	471.9/531.3	3.0 × 10^−172^	10
HGLibB_09799	CLCA2	18.9/9.3	845.6/510.1	3.7 × 10^−146^	11
HGLibB_37031	PLEKHA6	49.3/2.1	391.1/577.1	5.7 × 10^−144^	12
HGLibA_56902	ZNF90	21.9/25.8	989.8/1188.4	5.5 × 10^−95^	13
HGLibA_06820	CA7	31.5/10.3	886.1/747.6	9.2 × 10^−106^	14
HGLibA_29915	MRPL18	10.7/55.7	1234.5/965.1	4.3 × 10^−90^	15
HGLibA_14727	EIF2AK2	25.5/13.4	746.2/778.1	1.6 × 10^−86^	16
HGLibB_44264	SLAMF7	238.1/408.7	13,929.1/11,287.1	1.3 × 10^−21^	17
HGLibA_61495	PER2	79.5/72.2	2574.1/3308.8	6.2 × 10^−39^	18
HGLibA_00885	ADAT3	16.6/13.4	729.8/405.5	4.6 × 10^−74^	19
HGLibA_25835	KRTAP9-3	22.5/29.9	1108.9/794.6	1.6 × 10^−56^	20
HGLibA_60473	PER1	42.1/18.5	1010.6/980.3	7.9 × 10^−53^	21
HGLibA_20486	GSTO1	30.3/6.2	512.4/444.3	8.1 × 10^−70^	22
HGLibB_45186	SLC51A	26.1/29.9	867.4/1028.5	1.6 × 10^−47^	23
HGLibA_36058	PER2	33.3/21.7	1025.9/779.3	5.0 × 10^−47^	24
HGLibA_05746	C1orf111	22.5/25.8	773.5/795.7	5.1 × 10^−47^	25

**Table 2:** Top 24 CRISPRa library enriched sgRNA upon TMZ treatment.

sgRNA Sequence	Gene	Control_count	Treatment_count	FDR	Rank
GTTATCACGGACAGGGAATA	MSANTD3	4.9/5.4	1289.4/1079.7	0	1
CCCGCACTCAAGGAGGTCTT	NFE2L2	241.1/555.1	55,614.1/69,280.1	0	2
GACGCCGGCCCGCAAGTGAC	GCLM	4.9/9.9	750.1/892.1	8.90 × 10^−291^	3
CGCAAGGCTGCCGCAGCCAA	HDAC4	9.7/7.8	991.2/829.8	2.60 × 10^−283^	4
CGGGGTGGCCTATCGCCGCC	HDAC4	9.7/6.1	653.6/730.7	6.30 × 10^−278^	5
CCCGCACTCAAGGAGGTCTT	NFE2L2	16.7/13.4	1376.3/1028.8	2.90 × 10^−75^	6
GCGGCAGGGGCCAGCCCGCC	UBE2E3	10.8/12.2	1029.5/795.2	3.00 × 10^−238^	7
GGCCAGGACAAAGAGCGCGG	UBE2E3	22.6/1.6	1013.1/877.5	1.70 × 10^−183^	8
CCGAGCAGCCGCCGCTGCTT	GDNF	7.3/7.9	519.2/407.1	3.30 × 10^−159^	9
CCCTGACCCGGGTGTCACCG	EXTL3	10.7/12.2	662.1/691.5	3.50 × 10^−130^	10
TACACACATTTCGTGGGGTG	USP46	7.7/5.6	403.9/329.3	1.90 × 10^−^73	11
GAATGAATGTTATCACGGAC	MSANTD3	3.4/11.1	480.2/309.1	7.70 × 10^−127^	12
CCGAGCAGCCGCCGCTGCTT	GDNF	23.9/7.9	906.6/770.6	6.20 × 10^−120^	13
CATATGACTCAGCACATGAC	MSANTD3	29.5/15.9	1201.4/934.4	7.20 × 10^−114^	14
AGTACTGGGAGCGATTCTTG	GNAS	34.8/19.8	1140.1/1025.1	2.40 × 10^−59^	15
CGGGGTGGCCTATCGCCGCC	HDAC4	32.46/15.4	932.8/847.8	1.30 × 10^−83^	16
GAATGAATGTTATCACGGAC	MSANTD3	13.3/18.3	587.3/590.1	3.50 × 10^−83^	17
GGGCCGGGCGCCGGGGATTG	EXTL3	4.8/10.8	591;1/544.6	3.60 × 10^−78^	18
CGCCTTCTCCGGGCGCATCC	BOK	22.9/16.7	592.9/795.2	2.40 × 10^−51^	19
AGCAGTTCAACTTCCTATTA	FZD6	8.6/15.9	479.4/364.5	5.10 × 10^−77^	20
AAAGCCGGCGGCGGCGTCCA	ABHD17C	24.8/35.4	890.3/954.7	7.00 × 10^−59^	21
AGCGGCCCCTGGGCCAAGCC	ABCC3	39.6/36.2	1030.9/1233.1	3.30 × 10^−68^	22
CGTCGAGGGGGGCAAGCGCC	CERS4	19.8/13.7	501.1/297.3	8.00 × 10^−72^	23
CGGCGTGTCGCGTCAGACGC	POLK	52.5/35.7	893.9/1099.3	1.40 × 10^−69^	24

## Supplementary Materials

The following are available online at https://www.mdpi.com/2073-4409/9/12/2573/s1, Table S1: List of CRISPRko library enriched genes; Table S2: List of CRISPRa library enriched genes.
